# An analysis of clinical characteristics and prognosis of endometrioid ovarian cancer based on the SEER database and two centers in China

**DOI:** 10.1186/s12885-023-11048-1

**Published:** 2023-07-01

**Authors:** Shuangfeng Chen, Huaiwu Lu, Shan Jiang, Min Li, Haiyan Weng, Jing Zhu, Tianjiao Zhang, Yingying Wang, Weidong Zhao, Dabao Wu, Zhen Shen, Zhenye Yang, Ying Zhou

**Affiliations:** 1 Department of Obstetrics and Gynecology, Provincial Hospital Affiliated to Anhui Medical University, Hefei, Anhui China; 2 Department of Gynecologic Oncology, Sun Yat-Sen Memorial Hospital, Sun Yat-Sen University, Guangzhou, Guangdong China; 3 Department of Obstetrics and Gynecology, Core Facility Center, The First Affiliated Hospital of USTC, Division of Life Sciences and Medicine, University of Science and Technology of China, Hefei, Anhui 230001 China; 4 Department of Pathology, The First Affiliated Hospital of USTC, Division of Life Sciences and Medicine, University of Science and Technology of China, Hefei, Anhui 230001 China; 5 Hefei National Laboratory for Physical Sciences at Microscale, CAS Key Laboratory of Innate Immunity and Chronic Disease, The First Affiliated Hospital of USTC, Division of Life Sciences and Medicine, University of Science and Technology of China, Hefei, Anhui China

**Keywords:** Endometrioid ovarian carcinoma, Clinical characteristics, Prognostic factors, Prognostic model

## Abstract

**Purpose:**

To assess the clinical characteristics and the risk factors related to the unfavorable prognosis of endometrioid ovarian carcinoma (EOVC) based on data from the Surveillance, Epidemiology, and End Results (SEER) database and two clinical centers in China.

**Methods:**

Data were extracted from the SEER database and two clinical centers in China (2010 ~ 2021), 884 cases and 87 patients with EOVC were selected, respectively. Overall survival (OS) and progression-free survival (PFS) were compared among the different groups using Kaplan–Meier analysis. The Cox proportional-hazards model was used to identify independent prognostic factors related to EOVC. A nomogram was constructed based on the risk factors of the SEER database affecting prognosis and the discrimination and calibration of the nomogram were evaluated by C-index and calibration curves.

**Results:**

The average age at diagnosis of patients with EOVC in the SEER database and two centers in China was 55.77 ± 12.40 years and 47.14 ± 11.50 years, 84.7% and 66.6% of them were diagnosed at FIGO stage I ~ II, respectively. In the SEER database, age over 70 years, advanced FIGO stage, tumor grade 3, only unilateral salpingo-oophorectomy were independent risk factors of unfavorable prognosis. In two clinical centers in China, 27.6% of EOVC patients were diagnosed with synchronous endometriosis. Advanced FIGO stage, HE4 > 179 pmol/L and bilateral ovarian involvement significantly correlated with poor OS and PFS in Kaplan–Meier analysis. Body mass index (BMI) < 19.34 kg/m^2^ was an independent risk factor relating to OS and PFS. Additionally, C-index of internal and external verification for the nomogram were 0.812 and 0.754 respectively, revealing good accuracy and clinical applicability.

**Conclusions:**

Most patients were diagnosed at early stage, low grade and had better prognosis. Asian/Pacific Islander and Chinese diagnosed with EOVC were more likely to be younger than whites and blacks. Age, tumor grade and FIGO stage (SEER database) and BMI (two centers) are independent prognostic factors. HE4 appears to be more valuable in prognostic assessment compared with CA125. The nomogram had good discrimination and calibration for predicting prognosis, providing a convenient and reliable tool for clinical decision-making for patients with EOVC.

**Supplementary Information:**

The online version contains supplementary material available at 10.1186/s12885-023-11048-1.

## Introduction

Endometrioid ovarian cancer (EOVC) accounts for approximately 10% ~ 13% of epithelial ovarian cancer (EOC) and has clinical and biological difference compared with other pathological subtypes [1–3]. The incidence of EOVC in non-Hispanic, Hispanic and Asian populations is about 8%, 9.6% and 11%, respectively [4]. Domestic and foreign research generally believed that endometriosis, genes mutation, imbalance of female reproductive tract microenvironment, delayed menopause and menopausal hormone replacement, obesity and other factors can increase the risk of EOVC [5–7]. EOVC is one of endometriosis-associated ovarian cancer, the risk of which may increase 2.32 times if a patient has endometriosis [8]. Despite that, origin and pathogenesis of EOVC have not been revealed in detail, we still need lots of basic and clinical studies to explore.

Most patients with EOVC are diagnosed at an early stage, and the age of onset tends to be younger, with a better prognosis than that of high-grade serous cancer (HGSOC) and clear cell carcinoma (OCCC) [9]. CA125 and HE4 are tumor markers commonly used in clinical diagnosis and identification of EOC, but they all have a certain false positive rate. Moreover, in patients with EOVC, CA125 elevation is not as significant as in HGSOC, and about a quarter of patients had no abnormalities in CA 125 levels [10]. Study showed that HE4 has high sensitivity and specificity in diagnosis for EOC, with great potential in early patient diagnosis [11], but HE4 whether EOVC diagnostic and therapeutic efficacy monitoring is more advantageous still lack corresponding clinical evidence.

The Surveillance, Epidemiology, and End Results (SEER) database is currently the largest publicly available cancer statistics database that includes approximately 30% of the United States population [12]. In this study, we hope to evaluate clinical characteristics and explore risk factors affecting the prognosis of EOVC patients registered in the SEER database and two centers in China in order to provide some reference for EOVC diagnosis, decision making and prognosis assessment.

## Material and methods

### Study cohort

In this retrospective study, we obtained permission to access the SEER database and extracted data of 884 EOVC patients from Incidence-SEER Research Data, 9 Registries, Nov 2020 Sub (1975 ~ 2018) using the SEER*Stat software, version 8.3.8. Inclusion criteria are as follows: (1) the primary site of malignant tumor is restricted as "ovary"; (2) the pathological subtype is endometrioid carcinoma (ICD-O-3: 8380/3 Endometrioid carcinoma); (3) complete clinical, surgical, pathological, and follow-up data. Exclusion criteria are as follows: (1) the origin of the tumor is uncertain; (2) the patients who have not received surgical treatment; (3) a large number of cases with incomplete data indicators. Variables collected from the SEER database included age at diagnosis, race, origin recode, pathologic grade, SEER summary stage 2000, American Joint Committee on Cancer (AJCC) 7th staging, types of surgery, laterality, the number of lymph nodes (LN) resected, regional LN status, CS mets -brain, CS mets-liver, CS mets-lung, tumor size, CA125 status, follow-up status and survival time. The International Federation of Gynecology and Obstetrics (FIGO) staging is commonly used internationally for EOC, therefore we converted AJCC 7th staging into FIGO staging according to the National Comprehensive Cancer Network (NCCN) guidelines (Table S1) [13].

Equally, we also collected information on 87 patients diagnosed with primary EOVC between 2010 and 2021 at the First Affiliated Hospital of USTC and Sun Yat-Sen Memorial Hospital of Sun Yat-Sen University. Inclusion criteria are as follows: (1) the patients with histologically confirmed EOVC by pathologists who had undergone comprehensive staging surgery or primary cytoreductive surgery with adjuvant chemotherapy; (2) complete clinical information and pathological information; (3) exempt informed consent. Exclusion criteria are as follows: (1) the patients with synchronous ovary and endometrium carcinoma cannot identify the origin of the tumor; (2) the patients with incomplete clinical data; (3) the patients receiving adjuvant chemotherapy and radiotherapy before surgery; (4) the patients who in other hospital with unknown treatment conditions. Clinical data extracted includes demographic and pathologic characteristics, pre-operative biomarkers, surgery, chemotherapy regimen and course, survival time, and status at last follow-up. All clinical information was anonymized before analysis and this study was approved by the medical research ethics committees of the First Affiliated Hospital of University of Science and Technology of China (Ethics Approval No.2021-KY185). Detailed screening of all the cases included in this retrospective study is shown in Figure S1.

### Treatment and follow-up

Eight hundred and eighty-four patients with EOVC in the SEER database received initial surgical treatment. The types of surgery including unilateral salpingo-oophorectomy ± hysterectomy, bilateral salpingo-oophorectomy ± hysterectomy, salpingo-oophorectomy & omentectomy ± hysterectomy, and cytoreductive surgery. No chemotherapy information was obtained from the SEER database.

Eighty-seven patients from two centers in China received comprehensive staging surgery or primary debulking surgery. Only 14 patients with FIGO stage I were followed up after surgery, and the remaining patients received paclitaxel combined with platinum-based chemotherapy for 4 ~ 6 cycles after surgery. After treatment, all patients underwent pelvic examination and evaluation of tumor markers at each visit. If necessary, ultrasound, CT, or MRI were performed. Recurrence was defined as histologic evidence by tumor biopsy or fine-needle biopsy and/or the appearance of new lesions on imaging. Overall survival (OS) was defined as the time from initial diagnosis to the time of death by any cause or the last follow-up date, progression-free survival (PFS) was defined as the length of time during and after the treatment that a patient lives with the disease without it getting worse.

### Statistical analysis

All statistical analyses were performed using IBM SPSS, version 26.0 and GraphPad Prism, version 6.0. Baseline characteristics were summarized into means or medians, or counts, or percentages, as appropriate. In the analysis using Kaplan–Meier curves, some variables were classified by cut-off value and the differences between subgroups were evaluated by log-rank test. The chi-square test was used to assess associations between categorical variables. Hazard ratios (HR) were determined using univariate and multivariate Cox proportional-hazards models. *P*-value of less than 0.05 was considered statistically significant and P-value of less than 0.01 was considered highly significant. The nomogram model was constructed, and C-index and calibration curves were calculated using the R Statistical Software, version 4.1.1.

## Results

### SEER Database

#### Clinical characteristics (Table 1)

**Table 1:** Baseline characteristics of 884 EOVC patients in the SEER database

Variables	SEER database (NO.)	%
Total	884	
**Age of diagnosis**	55.77 ± 12.40^**a**^	
≤ 50y	311	35.2
51 ~ 60y	282	31.9
61 ~ 70y	176	19.9
> 70y	115	13.0
**Race**		
White	696	78.7
Black	50	5.7
Asian/Pacific Islander	119	13.5
Other	19	2.1
**Origin**		
Non-Spanish-Hispanic-Latino	800	90.5
Spanish-Hispanic-Latino	84	9.5
**Laterality**		
Unilateral	757	85.6
Bilateral	125	14.1
Unknown	2	0.2
**Tumor Size**		
< 1 cm	25	2.8
1 ~ 4 cm	131	14.8
5 ~ 10 cm	235	26.6
> 10 cm	413	46.7
Unknown	80	9.1
**Summary stage 2000**		
Localized	364	41.2
Regional	407	46.0
Distant	113	12.8
**Grade**		
G1	342	38.7
G2	340	38.5
G3	170	19.3
G4	32	3.6
**FIGO stage**		
I	598	67.6
II	151	17.1
III	104	11.8
IV	31	3.5
**FIGO stage**		
I ~ IIIB	802	90.7
IIIC ~ IV	72	8.1
Unknown	10	1.1
**Regional LN resected**		
None/Unknown	173	19.6
1 ~ 3	77	8.7
4 or more	634	71.7
**Types of surgery**		
USO (hys/nonhys)	31	3.4
BSO (hys/nonhys)	204	23.1
SO and Ome (hys/nonhys)	401	45.4
Debulking	236	26.7
Other	12	1.4
**Regional LN status**		
Positive	50	5.7
Negative	661	74.8
Unchecked	173	19.5
**CA125 status**		
Positive	539	61.0
Negative	112	12.7
Unknown	233	26.3
**CS mets**		
Liver	12	1.4
Lung	4	0.5
Brain	1	0.1
**Status**		
Alive	742	83.9
Dead	142	16.1

^a^: mean ± stdev, *LN* Lymph node, *USO (hys/nonhys)* Unilateral salpingo-oophorectomy, *BSO (hys/nonhys)* Bilateral salpingo-oophorectomy, *SO&Ome (hys/nonhys)* Salpingo-oophorectomy & omentectomy, *Debulking* Cytoreductive surgery

The mean age at diagnosis of 884 patients with EOVC is 55.77 ± 12.40 years (range, 21 ~ 85 + years). The distribution of age category was 35.2%, 31.9%, 19.9% and 13.0%, for those aged 50 years or younger, 51 to 60 years, 61 to 70 years, and those older than 70 years, respectively. Only 72 (8.1%) patients were diagnosed with advanced-stage cancer (FIGO IIIC ~ IV) and 802 patients (90.7%) had early-stage (FIGO IA ~ IIIB) cancer. There were 50 (5.7%) patients diagnosed with regional LN positive and 539 (61.0%) patients showed abnormal CA125 status. And 17 patients had distant metastases to the brain (1 case), lung (4 cases) or liver (12 cases). Finally, 142 out of 884 patients died and 107 of these deaths were attributed to the disease.

#### Prognosis

**Fig. 1:**
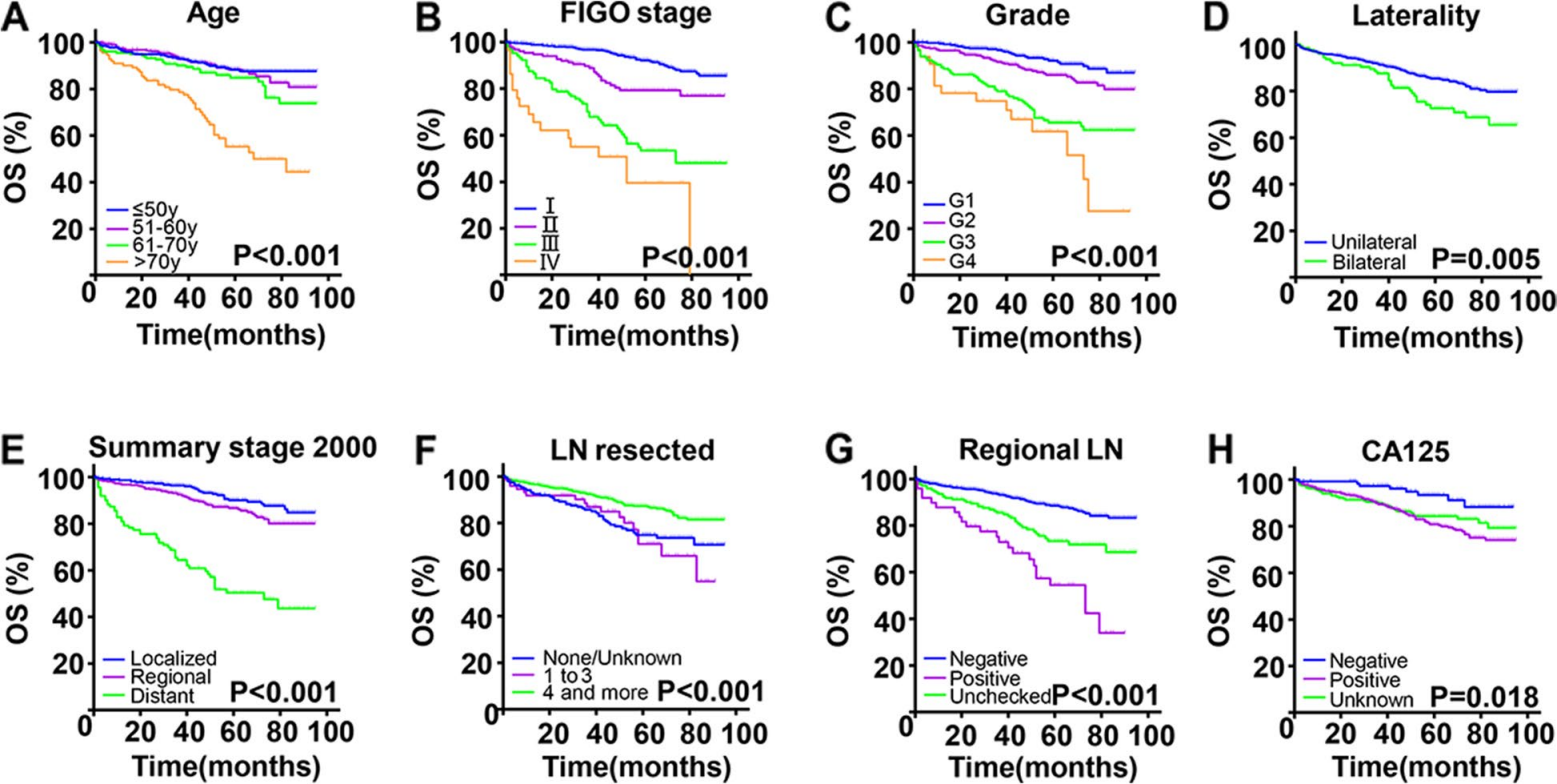
Kaplan–Meier curves for overall survival in 884 patients with EOVC. Grouped by age (**A**), FIGO stage (**B**), tumor grade (**C**), laterality (**D**), summary stage 2000 (**E**), the number of LN resected (**F**), regional LN status (**G**), CA125 status(**H**)

The median OS of this cohort was 52 months. The Kaplan–Meier analysis (Fig. 1) showed that age (*P* < 0.001), FIGO stage (*P* < 0.001), grade (*P* < 0.001), SEER summary stage 2000 (*P* < 0.001), the number of LN resected (*P* < 0.001) and regional LN status (*P* < 0.001) were highly significantly associated with OS. Factors with *P* < 0.01 in univariate Cox analysis were further included in multivariate analysis, showing that age (> 70y), tumor grade (G3) and FIGO stage (III ~ IV) and types of surgery (unilateral salpingo-oophorectomy) were significant independent risk factor (Table 2).

**Table 2:** Univariate and multivariate Cox analysis of the overall survival of 884 patients in the SEER database

Variables	**Overall survival** (Univariable cox analysis)		**Overall survival** (Multivariable cox analysis)		
	HR (95% CI)	*P* value	HR (95% CI)	*P* value	*P* value^a^
**Age of diagnosis**					
≤ 50y	Ref		Ref	-	-
51 ~ 60y	1.162(0.714 ~ 1.889)	0.546	1.276(0.716 ~ 2.274)	0.408	0.424
61 ~ 70y	1.740(1.057 ~ 2.863)	0.029	1.549(0.837 ~ 2.868)	0.164	0.193
> 70y	5.073(3.232 ~ 7.962)	**< 0.001**	5.064(2.793 ~ 9.182)	**< 0.001**	**< 0.001**
**Laterality**					
Unilateral	Ref	-	Ref	-	
Bilateral	1.708(1.146 ~ 2.546)	**0.009**	0.699(0.408 ~ 1.198)	0.193	0.165
**Summary stage 2000**					
Localized	Ref	-	Ref	-	-
Regional	1.579(1.027 ~ 2.428)	0.037	0.699 (0.335 ~ 1.457)	0.339	0.261
Distant	6.841(4.426 ~ 10.574)	** < 0.001**	0.758(0.174 ~ 3.305)	0.713	0.433
**Grade**					
G1	Ref	-	Ref	-	-
G2	1.980(1.231 ~ 3.186)	**0.005**	1.957(1.045 ~ 3.664)	0.036	0.054
G3	4.597(2.875 ~ 7.351)	**< 0.001**	3.248(1.652 ~ 6.386)	**0.001**	**0.002**
G4	7.351(3.836 ~ 14.088)	**< 0.001**	3.008(1.174 ~ 7.709)	0.022	0.086
**FIGO stage**					
I	Ref	-	Ref	-	-
II	2.463(1.550 ~ 3.912)	**< 0.001**	2.991(1.426 ~ 6.275)	**0.004**	**0.005**
III	6.281(4.197 ~ 9.400)	**< 0.001**	5.361(1.531 ~ 18.772)	**0.009**	0.016
IV	12.531(7.371 ~ 21.302)	**< 0.001**	10.483(2.610 ~ 42.104)	**0.001**	**0.002**
**FIGO stage**					
I ~ IIIB	Ref	-	Ref	-	-
IIIC ~ IV	5.808(3.987 ~ 8.462)	**< 0.001**	1.033(0.398 ~ 2.683)	0.947	0.955
**Types of surgery**					
Debulking	Ref	-	Ref	-	-
SO&Ome	0.406(0.277 ~ 0.595)	**< 0.001**	1.011(0.068 ~ 1.680)	0.968	0.972
USO	0.590(0.237 ~ 1.470)	0.257	5.889(2.047 ~ 16.938)	**0.001**	**0.001**
BSO	0.392(0.242 ~ 0.633)	**< 0.001**	0.971(0.494 ~ 1.907)	0.931	0.934
**The number of LN resected**					
None/Unknown	Ref	-	Ref	-	-
1 ~ 3	1.163(0.679 ~ 1.993)	0.582	0.396(0.076 ~ 2.060)	0.271	0.368
4 or more	0.510(0.353 ~ 0.738)	**< 0.001**	0.303(0.066 ~ 1.397)	0.126	0.221
**Regional LN status**					
Negative	Ref	-	Ref	-	-
Positive	4.571(2.867 ~ 7.289)	**< 0.001**	0.987(0.420 ~ 2.319)	0.976	0.978
Unchecked	2.265(1.555 ~ 3.299)	**< 0.001**	0.384(0.079 ~ 1.856)	0.234	0.315
**CA125 status**					
Negative	Ref	-	Ref	-	-
Positive	2.833(1.378 ~ 5.824)	**0.005**	2.012(0.917 ~ 4.415)	0.081	0.094
**Race**					
White	Ref	-			
Black	1.494 (0.824 ~ 2.710)	0.186			
Asian/Pacific Islander	0.860 (0.501–1.473)	0.582			
Other	0.622(0.154 ~ 2.519)	0.506			
**Origin**					
Non-Spanish	Ref	-			
Spanish	0.838(0.453 ~ 1.550)	0.572			
**Tumor size**					
< 1 cm	Ref	-			
1 ~ 4 cm	1.755(0.403 ~ 7.635)	0.453			
5 ~ 10 cm	1.851(0.443 ~ 7.735)	0.399			
> 10 cm	2.513(0.617 ~ 10.242)	0.199			

*HR* Hazard ratio, ^a^: based on 5000 bootstrap samples

#### Construction and inner validation of nomogram

**Fig. 2:**
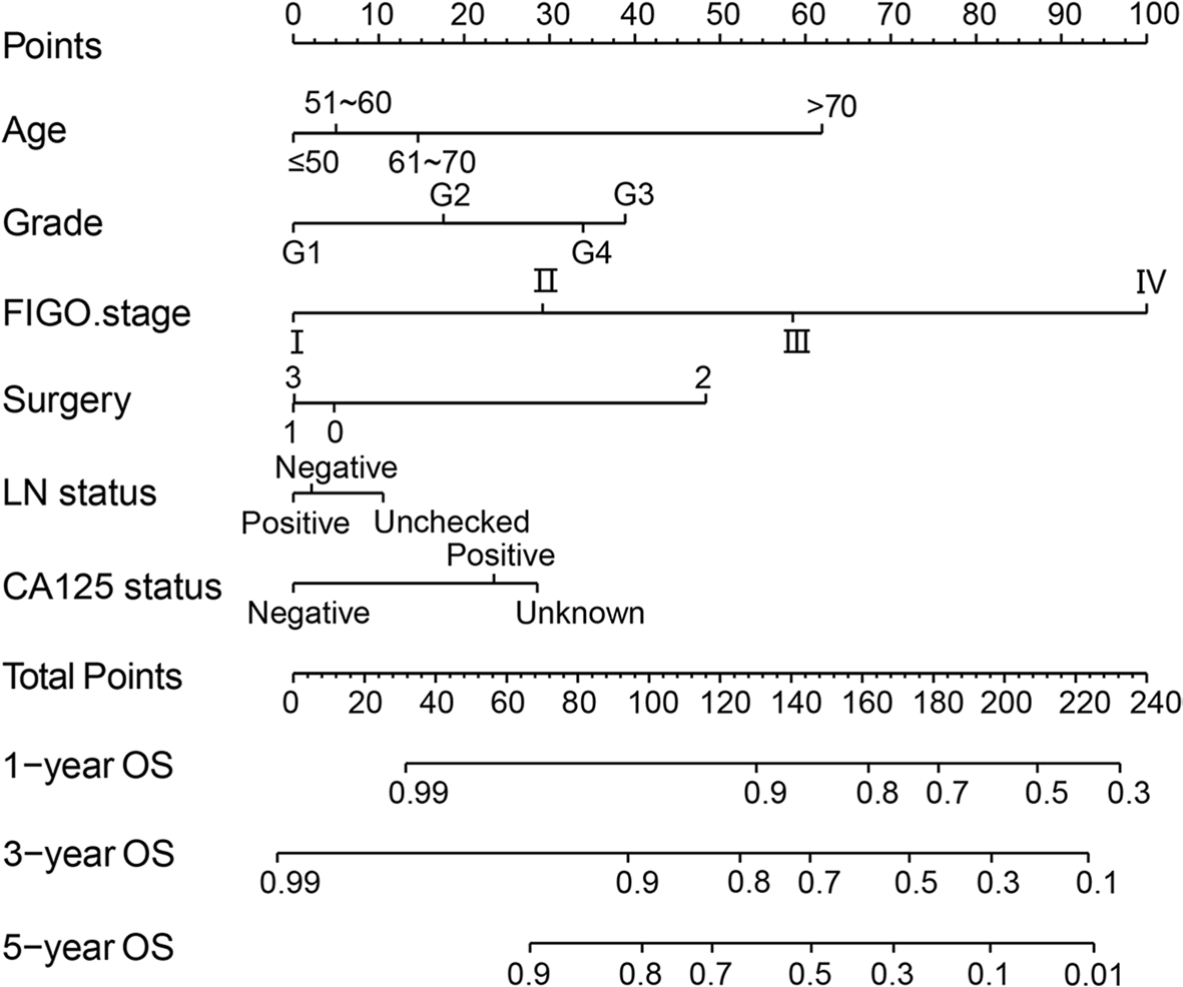
Prognostic nomogram of 1-, 3-, 5-years overall survival for patients with EOVC. Surgery (0: Debulking Surgery; 1: SO and Ome; 2: USO; 3: BSO) [SO and Ome (hys/nonhys): salpingo-oophorectomy & omentectomy; USO (hys/nonhys): unilateral salpingo-oophorectomy; BSO (hys/nonhys): bilateral salpingo-oophorectomy]

Based on results of analysis, age, tumor grade, FIGO stage, types of surgery, regional LN status and CA125 status were combined to construct nomogram model in order to predict the one-year, three-year, and five-year survival probabilities of patients (Fig. 2). The C-index used to assess the predictive accuracy of the nomograms were 0.812 (95% CI, 0.793 ~ 0.831) for OS in internal validation. Using the bootstrap self-sampling method, the calibration curves of one-year, three-year and five-year OS prediction were drawn (Figure S2).

### Two clinical centers in China

#### Clinical characteristics (Table 3)

**Table 3:** Baseline characteristics of 87 EOVC patients in the two clinical centers in China

Variables	No	%
**Total**	87	
**Age of diagnosis**	47.14 ± 11.50^**a**^	
≤ 50y	50	57.5
51 ~ 60y	28	32.2
> 60y	9	10.3
**Menopause**		
Pre-	33	37.9
Post-	54	62.1
**Body mass index (kg/m** ^**2**^ **)**		
< 18.5	8	9.2
18.5 ~ 23.9	45	51.7
24 ~ 27.9	13	14.9
≥ 28	5	5.8
Unknown	16	18.4
**Laterality**		
Unilateral	57	65.5
Bilateral	22	25.3
Unknown	8	9.2
**FIGO stage**		
I	51	58.6
II	7	8.0
III	26	29.9
IV	3	3.5
**Grade**		
G1	16	18.4
G2	40	46.0
G3	21	24.1
G4	1	1.2
Unknown	9	10.3
**Endometriosis**	24	27.6
**LN positive**	11	12.6
**Adenomyosis**	4	4.6
**Comorbidities**		
Diabetes	5	5.7
Hypertension	8	9.2
Venous thromboembolism	1	1.1
Breast cancer history	1	1.1
Connective tissue disease	2	2.3
Thyroid disease	2	2.3
**Preoperative laboratory test**		
**CA125(U/ml)**		
Negative (< 35)	9	10.3
Positive (≥ 35)	74	85.1
35 ~ 99	19	21.8
100 ~ 499	26	29.9
≥ 500	29	33.3
Unknown	4	4.6
**HE4 (pM)**		
Negative (< 70)	19	21.8
Positive (≥ 70)	56	64.4
70 ~ 499	39	44.8
≥ 500	17	19.5
Unknown	12	13.8
**Cytoreduction**		
R0	74	85.1
R1 ~ R2	6	6.9
Unknown	7	8.0
**Status**		
Alive	59	67.8
Dead	10	11.5
Loss of follow up	18	20.7

^a^: mean ± stdev

The mean age at diagnosis of 87 EOVC patients was 47.14 ± 11.50 years (range, 25 ~ 77 years). Majority of patients (65/87, 74.7%) were diagnosed with early-stage disease (FIGO IA ~ IIIB). The CA125 assay was performed in 83 patients and the median level was 235.8 U/mL (range, 10.78 ~ 21178 U/mL) and of these, only 9 (10.3%) patients had a negative CA125 level. The median level of HE4 was 178.8 pmol/L (range 39.48 ~ 9999 pmol/L) and the proportions of negative expression were 21.8%. Of the 87 patients, 24 had co-existing endometriosis (27.6%).

There were 65 (74.7%) patients and 22 (25.3%) patients who received complete staging surgery and cytoreductive surgery, respectively (Table S2). 11 patients (12.6%) had LN metastasis. Among patients with advanced EOVC, optimal cytoreduction was achieved in half of the patients (11/22). Adjuvant chemotherapy with paclitaxel and platinum was performed in 73 patients (83.9%). Of these, 43 (49.4%) patients completed the initial chemotherapy within two weeks of surgery and a total of 62 (71.3%) patients had completed chemotherapy within four weeks of surgery. The remaining 11 patients did not accept chemotherapy within the four weeks due to anemia, infection, or personal reasons.

In the group of 24 patients with co-existing endometriosis, the mean age at diagnosis was 47.25 ± 11.13 years. A comparison of the clinical characteristics of patients with and without co-existing endometriosis is presented in Table S3; except for laterality (*P* = 0.044), there is no statistical significance of other indicators between the two groups.

#### Prognosis

Twelve patients eventually relapsed after receiving at least six cycles of taxanes plus platinum following staging or primary cytoreductive surgery. After recurrence, one patient underwent re-cytoreductive surgery and chemotherapy and seven patients only underwent chemotherapy. Eventually, ten patients died of the disease (Table S4).

**Fig. 3:**
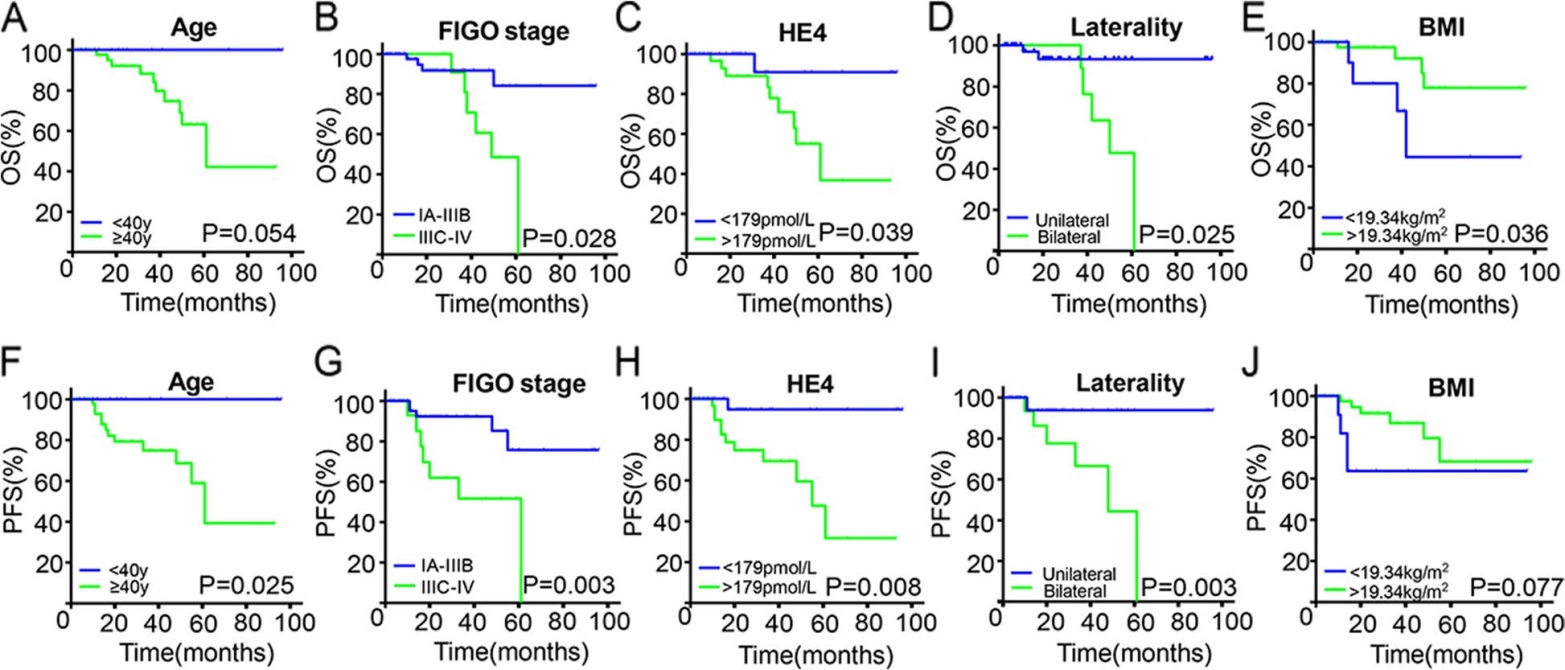
Kaplan–Meier curves of overall survival and progression-free survival of 69 patients in our centers. Grouped by age, FIGO stage, HE4 level, laterality and BMI

A total of 69 (79.3%) patients were followed-up continuously. Among these, 50 were followed up for more than one year, 29 for more than three years, and only eight for more than five years. Kaplan–Meier survival curves (Fig. 3) showed that FIGO stage (*P* = 0.028), HE4 level (*P* = 0.039), laterality (*P* = 0.025) and Body mass index (BMI) (*P* = 0.036) were certain associated with OS in patients with EOVC; age (*P* = 0.025), FIGO stage (*P* = 0.003), HE4 level (*P* = 0.008) and laterality (*P* = 0.003) were certain associated with PFS in patients with EOVC. Factors with *P* < 0.1 in univariate Cox analysis of OS were further included in multivariate analysis, showing that BMI (*P* = 0.020), laterality (*P* = 0.028), and advanced-stage tumor (*P* = 0.032) had statistical significance. Similarly, BMI (*P* = 0.018) had statistical significance in the multivariate bootstrap analysis of PFS (Table 4).

**Table 4:** Univariate and multivariate Cox analysis of prognostic factors in 69 patients with EOVC

	**Overall survival** (Univariable cox analysis)			**Overall survival** (Multivariable cox analysis)		**Progression free survival** (Univariable cox analysis)			**Progression free survival** (Multivariable cox analysis)	
	HR	*P* value	*P* value^a^	*P* value	*P* value^a^	HR	*P* value	*P* value^a^	*P* value	*P* value^a^
Age^b^	37.041 (0.082 ~ 16,737.327)	0.247	**0.001**	0.968	0.625	37.822 (0.178 ~ 8054.851)	0.184	**0.001**	0.977	0.654
Menopause	1.282 (0.361 ~ 4.559)	0.701	0.703			1.258 (0.397 ~ 3.988)	0.697	0.692		
BMI^b^	0.249 (0.061 ~ 1.010)	0.052	**0.014**	0.086	**0.020**	0.336 (0.094 ~ 1.200)	0.093	0.081	0.366	**0.018**
Laterality	5.454 (1.041 ~ 28.583)	**0.045**	**0.021**	0.195	**0.028**	7.889 (1.581 ~ 39.373)	**0.012**	**0.005**	0.287	0.142
FIGO stage^c^	3.829 (1.061 ~ 13.817)	**0.040**	**0.009**	0.315	**0.032**	4.895 (1.544 ~ 15.525)	**0.007**	**0.001**	0.913	0.257
Grade^d^	2.631 (0.704 ~ 9.826)	0.150	0.101			1.957 (0.596 ~ 6.425)	0.268	0.282		
LN metastasis	1.383 (0.172 ~ 11.094)	0.760	0.779			1.155 (0.146 ~ 9.135)	0.891	0.909		
CA125 level^b^	2.968 (0.809 ~ 10.892)	0.101	0.069	0.449	0.067	2.361 (0.704 ~ 7.915)	0.164	0.161		
CA19-9 level^b^	5.419 (0.676 ~ 43.411)	0.111	0.061	0.670	0.464	0.330 (0.071 ~ 1.534)	0.157	0.073	0.734	0.553
HE4 level^b^	6.640 (0.834 ~ 52.853)	0.074	0.058	0.961	0.642	9.593 (1.235 ~ 74.521)	**0.031**	**0.025**	0.973	0.729
Albumin^b^	1.858 (0.337 ~ 10.178)	0.478	0.411			1.505 (0.291 ~ 7.778)	0.626	0.622		
Ascites (2000 ml)	0.905 (0.112 ~ 7.297)	0.925	0.932			2.041 (0.439 ~ 9.475)	0.363	0.186		
Endometriosis	0.451 (0.057 ~ 3.571)	0.451	0.358			0.332 (0.043 ~ 2.580)	0.292	0.178		
Interval day (14 days)	0.351 (0.073 ~ 1.694)	0.192	0.117			0.480 (0.127 ~ 1.815)	0.280	0.248		
Cytoreduction level	2.693 (0.308 ~ 23.525)	0.370	0.164			0.097 (0.017 ~ 0.537)	**0.008**	**0.001**	0.255	0.057

^a^: based on 5000 bootstrap samples

^b^: divide by cut-off value

^c^: I ~ IIIB vs IIIC ~ IV

^d^: grade1 ~ 2 vs grade 3

We also validated the nomogram using the data from our centers and the C-index of the nomograms were 0.754 for OS in external validation.

#### Comparison with the SEER database

The distribution of age, FIGO stage and grade of EOVC between the two cohorts is presented in Figure S3. The percentage of patients aged 51 to 60 years was higher in the two cohorts. The proportion of patients with G3 was 24.0% in Chinese samples, which is slightly higher than that of the SEER database. The percentage of Chinese patients with advanced-stage tumors especially those with FIGO stage III is relatively high.

**Fig. 4:**
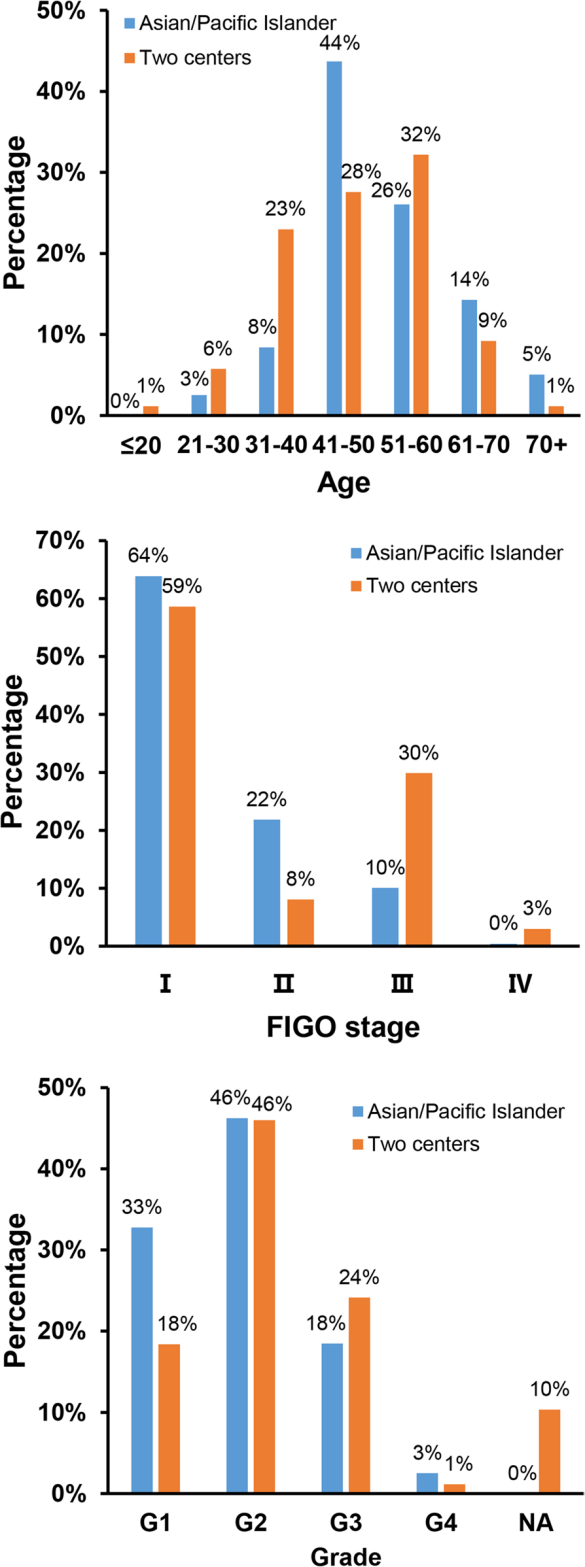
Distribution of age, FIGO stage and grade of EOVC patients of Asian/Pacific Islander and our centers

The population of SEER database was mainly divided into whites, blacks and Asian/Pacific Islander, and their average age of onset was 56.7 years old, 57.8 years old and 50.0 years old respectively. In contrast, the average age of onset in Asian/Pacific Islander is much younger and more similar to that of patients in our centers. The concentrated age of onset of Asian/Pacific Islander and Chinese samples is 41 to 50 years old (44%) and 51 to 60 years old (32%), respectively. However, the incidence of EOVC in our centers is significantly higher in people between 31 and 40 years of age than in Asian/Pacific Islander. Although the mean age at diagnosis was younger in our centers, the proportion of patients with FIGO III ~ IV stage was significantly higher than that of SEER database and Asian/Pacific Islander (Fig. 4).

## Discussion

EOVC, as a special subtype of EOC, has significant geographical and ethnic differences in incidence. Higher rates of EOVC in Asian women have been documented in the United States and Eastern Asia, although reasons remain unknown [3, 9, 14]. Previously, two single-center studies from China had confirmed that patients with EOVC were significantly younger compared to other histological types [15, 16]. In our study, we found that the average age of EOVC patients in two centers was approximately 8 years younger than that of patients in SEER database (47.14 years vs 55.77 years). However, due to the diversity of races in SEER database, we further analyzed and found that the average age of EOVC of Asian/Pacific Islander was younger than that of whites and blacks. In terms of age stratification, Asian/Pacific Islander had the largest number of cases in the 41 ~ 50 age group, which may partly reflect that EOVC may occur at a younger age in Asians, including Chinese people. Meanwhile, the results from SEER database also found that age is an independent risk factor affecting the prognosis, but same conclusion was not drawn according to 87 EOVC patients from China. This may be related to the small samples we obtained, followed by differences in morbidity characteristics because of different populations, regions and health care measures, therefore, multicentric clinical studies are needed to analyze the distribution characteristics and incidence of EOVC patients in China in the future.

Endometriosis is thought to play a role in the pathogenesis of ovarian cancer, it’s estimated that patients with endometriosis have a fourfold and 2.32-fold increased risk of ovarian cancer and EOVC, respectively [8, 17]. In our study, we found that endometriosis occurred in approximately 27.6% of patients and was not an independent prognostic factor, which is in line with published articles [18, 19]. However, two large cohort studies based on the PALGA database and the Netherlands Cancer Registry revealed that a significantly higher incidence of EOVC was observed in women with endometriosis and a better survival presented in women with endometriosis who had stage I ~ II EOVC [20, 21]. In a study by Giovanna et al. [22], unilateral ovarian involvement was more frequently found in OCCC with endometriosis, but similar results in EOVC have not been reported. In our cohort, we observed that unilateral ovarian involvement was more common in EOVC with endometriosis than without (87.5% vs 57.1%, *P* = 0.044). If the role of endometriosis in the pathogenesis of EOVC is further elucidated, a proper risk model for patients with endometriosis can be developed to prevent the occurrence of EOVC.

Surgical combined with chemotherapy is the most-common strategy used to treatment EOVC. The NCCN guidelines recommend patients with high grade should undergo adjuvant chemotherapy after surgery regardless of FIGO staging. A study assessing the effect of surgical staging and adjuvant chemotherapy on survival in stage I, low grade EOVC patients found that patients with G2 had a significantly high recurrence rate, but adjuvant chemotherapy and staging lymphadenectomy didn’t improve survival [23]. In contrast, another retrospective study by Dimitrios et al. showed that patients with G2 stage I tumors could benefit from adjuvant chemotherapy, which isn’t recommended for G1 tumor [24]. On the other hand, research from Oseledchyk et al. found that the benefit of adjuvant chemotherapy is limited to patients with stage IC and G3 EOVC [25]. Moreover, a multicenter study from Europe demonstrated disease grade wasn’t of prognostic significance if restricted to early-stage disease [26]. The frequency of G1 ~ 2 and G3 in patients with EOVC was 84% ~ 97% and 3% ~ 16%, respectively [19, 27, 28]. Although the proportion of poorly differentiated patient is low, some of these are more likely to develop chemotherapy resistance when recurrence occurred, then both treatment and prognosis face great challenges. In our two centers, a total of 12 EOVC patients experienced disease progression or recurrence during follow-up and there were seven cases with advanced stage, eventually 10 patients died within 3 years of relapse.

LN are a common site of metastasis in patients with EOC and the therapeutic value of lymphadenectomy during debulking surgery is still under debate [29]. As latest NCCN guidelines described, systematic lymph dissection for early-stage patients can confer clinical benefits in staging and follow-up treatment, the removal of all visible lesions for advanced-stage patients is recommendation in surgery, including enlarged or suspected LN. According to previous research data, the rate of LN metastasis was about 6.1% ~ 29.6% in patients with FIGO stage I ~ II ovarian cancer, and more than 50% in advanced patients. The probability of LN metastasis occurring in early-stage EOVC patients is about 2.1% ~ 6.5% [30, 31], but there are various views as to whether LN resection can provide survival benefits. A Chinese single-center retrospective study showed that LN resection was an independent protective factor for recurrence after operation in patients with FIGO stage I EOVC [15]. Meanwhile, another large-scale clinical study from US also confirmed that lymphadenectomy is associated with favorable survival [31]. However, a study from Italy didn’t reach the same conclusion [32]. In the study, the rates of LN metastasis were 12.6% and 5.7% in two Chinese centers and SEER database and, moreover, patients with FIGO stage III ~ IV EOVC are more often lymph-metastasized. Notably, LN metastases in the SEER database were related to poor prognosis. However, analysis for two Chinese centers didn’t reach same results (*P* = 0.887).

We found 12 patients diagnosed with endometrioid carcinoma of the ovary and uterus. However, these patients were excluded from our study because of the uncertainty about tumor origin. Synchronous diagnosis of endometrioid ovarian carcinoma and endometrioid endometrial carcinoma (SEO-EEC) is largely documented. Patients with SEO-EEC can be classified into three groups: endometrial cancer with metastasis to the ovary, ovarian cancer with metastasis to the endometrium, or synchronous primary cancers [33, 34]. Women with synchronous primary cancers have better survival rates than those with single cancers with metastases [35, 36]. The accurate distinction between metastases and independent primary carcinoma mainly depends on pathologic features [37], such as stage, whether there is fallopian tube and myometrial invasion, unilateral or bilateral ovarian involvement, presence of atypical endometrial hyperplasia, presence of ovarian endometriosis, and follow-up status of patients, which is important. Moreover, molecular profiling is also helpful in the evaluation of primary or metastatic tumors, and can identify Lynch syndrome. Unfortunately, due to the limitations of basic healthcare insurance in China, molecular profiling wasn’t done as most patients were not willing to pay out-of-pocket for, thus it was difficult to identify metastasis from independent primary carcinoma. An accurate distinction is of great importance for prognostic and therapeutic assessment.

The study has a few limitations that should be noted. Firstly, the satisfaction of surgery and the sensitivity of adjuvant chemotherapy after surgery affect the overall therapeutic effect of EOVC patients to a large extent. Regrettably, the SEER database didn’t contain detailed postoperative chemotherapy information. The nomogram model constructed based on SEER database may have certain deviation in prognostic prediction on account of the lack of important adjuvant chemotherapy indicators, which can be further improved in subsequent prospective multi-center studies and include more effective indicators in order to build a prognostic prediction model for Chinese patients with EOVC. Secondly, the SEER database provided no information on BMI, HE4, CA19-9, cancer recurrence, and history of endometriosis, it wasn’t possible to compare more detailed differences in clinical characteristics between the two cohorts. Thirdly, the tolerance and severity of adverse reactions of patients in our centers to chemotherapy will affect the cycle and dose of chemotherapy and the therapeutic effect. Due to the limitation of the retrospective study, we did not include more factors affecting chemotherapy for prognostic evaluation, which may cause some offset in the results of the final analysis. We expect to conduct more clinical studies in subsequent studies on patients with ovarian cancer who are not sensitive to advanced chemotherapy. In addition, BMI was an independent prognostic factor in the analysis of data from the two centers in China. Owing to the nature of our study, we did not have information on the hip circumference or the waist-to-hip ratio, which might to be better predictors of all-cause mortality. CA125 and HE4 are important indictors for diagnosis and prognosis evaluation of EOC, it was found that different from HGSOC, HE4 may have greater predictive value for EOVC. Of course, this conclusion was only based on small sample size data, which lacks representation of the whole population, and needs to be verified by multi-center and large-sample clinical studies.

In conclusion, most EOVCs were diagnosed at an early stage, low grade and had better prognosis. The age of onset for EOVC may be younger in Asian/Pacific Islander and Chinese. The elder, high grade, advanced FIGO stage, only unilateral salpingo-oophorectomy, and BMI < 19.34 kg/m^2^ may be unfavorable risk factors for prognosis. In addition, HE4 and CA125, as prognostic indicator, has certain guiding significance. A prognostic nomogram was developed and validated to assist clinicians in evaluating prognosis of EOVC patients. In the future, more studies are needed to further explore the molecular pathogenesis and treatment strategies of EOVC, a special pathological type, so as to provide new directions and ideas for early screening and early diagnosis of EOVC and improvement of advanced poor prognosis.

## Supplementary Information

**Additional file 1: Table S1.** FIGO and AJCC staging system for epithelial ovarian carcinoma. **Table S2.** Surgical procedures and clinical outcomes of 87 patients with EOVC. **Table S3.** Comparison of EOVC patients with/without endometriosis in the two clinical centers in China. **Table S4.** Treatment and survival outcomes of the 12 patients with recurrence.

**Additional file 2:** **Figure S1.** Flowchart of the selection for patients with endometrioid ovarian carcinoma. **Figure S2.** The calibration curves of one-year, three-year and five-year overall survival of the nomogram. **Figure S3.** Distribution of age, FIGO stage and grade of EOVC patients of the SEER database (*N*=884) and our centers (*N*=87).

## Data Availability

We signed a Data Use Agreement (DUA) for the SEER data which states that SEER data cannot be re-released. The data of two China clinical centers analyzed during the current study are available from the corresponding author on reasonable request.

## Abbreviations

EOVC: Endometrioid ovarian carcinoma
EOC: Epithelial ovarian cancer
HGSOC: High-grade serous ovarian cancer
OCCC: Ovarian clear cell cancer
CA125: Carbohydrate antigen 125
HE4: Human epididymis protein4
SEER: The surveillance, epidemiology, and end results
AJCC: American joint committee on cancer
LN: Lymph node
FIGO: The international federation of gynecology and obstetrics
NCCN: National comprehensive cancer network
OS: Overall survival
PFS: Progression-free survival
HR: Hazard ratios
BMI: Body mass index
PALGA: Dutch nationwide registry of histopathology and cytopathology Netherlands cancer registry
MMR: Mismatch repair
IHC: Immunohistochemistry
TCGA: The cancer genome atlas
EC: Endometrial carcinoma
SEO-EEC: Synchronous endometrioid ovarian carcinoma and endometrioid endometrial carcinoma
USTC: University of Science and Technology of China
