# Supplementary material for: An analysis of clinical characteristics and prognosis of endometrioid ovarian cancer based on the SEER database and two centers in China
Source: BMC Cancer. 2023 Jul 1;23:608. doi: 10.1186/s12885-023-11048-1 (PMC10314552; doi:10.1186/s12885-023-11048-1)
Supplement: Supplementary file 1 — Additional file 1: Table S1. FIGO and AJCC staging system for epithelial ovarian carcinoma. Table S2. Surgical procedures and clinical outcomes of 87 patients with EOVC. Table S3. Comparison of EOVC patients with/without endometriosis in the two clinical centers in China. Table S4. Treatment and survival outcomes of the 12 patients with recurrence. [file 12885_2023_11048_MOESM1_ESM.zip › Additional file 1/Supplementary Tables Title.docx]

**Table S1**. FIGO and AJCC staging system for epithelial ovarian carcinoma.

**Table S2**. Surgical procedures and clinical outcomes of 87 patients with EOVC.

**Table S3**. Comparison of EOVC patients with/without endometriosis in the two clinical centers in China.

**Table S4**. Treatment and survival outcomes of the 12 patients with recurrence.
