# Supplementary material for: An analysis of clinical characteristics and prognosis of endometrioid ovarian cancer based on the SEER database and two centers in China
Source: BMC Cancer. 2023 Jul 1;23:608. doi: 10.1186/s12885-023-11048-1 (PMC10314552; doi:10.1186/s12885-023-11048-1)
Supplement: Supplementary file 1 — Additional file 1: Table S1. FIGO and AJCC staging system for epithelial ovarian carcinoma. Table S2. Surgical procedures and clinical outcomes of 87 patients with EOVC. Table S3. Comparison of EOVC patients with/without endometriosis in the two clinical centers in China. Table S4. Treatment and survival outcomes of the 12 patients with recurrence. [file 12885_2023_11048_MOESM1_ESM.zip › Additional file 1/Table S1-S4 .docx]

Table S1. FIGO and AJCC staging system for epithelial ovarian carcinoma.

| **FIGO stage** | **AJCC stage** | | |
| --- | --- | --- | --- |
|  | **T** | **N** | **M** |
| **I** | T1 | N0 | M0 |
| IA | T1a | N0 | M0 |
| IB | T1b | N0 | M0 |
| IC | T1c | N0 | M0 |
| **II** | T2 | N0 | M0 |
| IIA | T2a | N0 | M0 |
| IIB | T2b | N0 | M0 |
| **III** | - |  |  |
| IIIA1 | T1/T2 | N1 | M0 |
| IIIA2 | T3a | NX/N0/N1 | M0 |
| IIIB | T3b | NX/N0/N1 | M0 |
| IIIC | T3c | NX/N0/N1 | M0 |
| **IV** | Any T | Any N | M1 |
| IVA | Any T | Any N | M1a |
| IVB | Any T | Any N | M1b |

Table S2. Surgical procedures and clinical outcomes of 87 patients with EOVC.

|  | Total | % | FIGO stage | | | |
| --- | --- | --- | --- | --- | --- | --- |
|  |  |  | Ⅰ~ⅢB  (n=65) | % | ⅢC~Ⅳ  (n=22) | % |
| Residual disease |  |  |  |  |  |  |
| Satisfactory reduction | 74 | 85.1 | 63 | 96.9 | 11 | 50.0 |
| Unsatisfactory reduction | 6 | 6.9 | 2 | 3.1 | 4 | 18.2 |
| Unknown | 7 | 8.0 | 0 | 0 | 7 | 31.8 |
| Ascites (ml) |  |  |  |  |  |  |
| <500 | 69 | 79.3 | 55 | 84.6 | 14 | 63.6 |
| 500~2999 | 11 | 12.6 | 6 | 9.2 | 5 | 22.7 |
| ≥3000 | 7 | 8.0 | 4 | 6.2 | 3 | 13.6 |
| LN positive | 11 | 12.6 | 4 | 6.2 | 7 | 31.8 |
| Estimated blood loss (ml) |  |  |  |  |  |  |
| <100 | 19 | 21.8 | 17 | 26.1 | 2 | 9.1 |
| 100~799 | 50 | 57.5 | 38 | 58.5 | 12 | 54.5 |
| ≥800 | 10 | 11.5 | 6 | 9.2 | 4 | 18.2 |
| Unknown | 8 | 9.2 | 4 | 6.2 | 4 | 18.2 |
| Recurrence | 12 | 13.8 | 5 | 7.7 | 7 | 31.8 |
| Endpoint status |  |  |  |  |  |  |
| Alive | 59 | 66.7 | 50 | 76.9 | 9 | 40.9 |
| Cancer specific deaths | 10 | 11.5 | 4 | 6.2 | 6 | 27.3 |
| Loss to follow-up | 18 | 21.8 | 11 | 16.9 | 7 | 31.8 |

Table S3. Comparison of EOVC patients with/without endometriosis in the two clinical centers in China.

|  | With  (n=24) | | Without  (n=63) | | P value |
| --- | --- | --- | --- | --- | --- |
|  | No. | % | No. | % |  |
| Age (years) | 47.25± 12.13^a^ |  | 47.09±11.34^a^ |  | 0.243 |
| ≤50y | 11 | 45.8 | 39 | 61.9 |  |
| 51~60y | 11 | 45.8 | 17 | 27.0 |  |
| ＞60y | 2 | 8.3 | 7 | 11.1 |  |
| FIGO stage |  |  |  |  | 0.117 |
| ⅠA~ⅢB | 21 | 87.5 | 45 | 71.4 |  |
| ⅢC~Ⅳ | 3 | 12.5 | 18 | 28.6 |  |
| Grade |  |  |  |  | 0.177 |
| G1/2 | 13 | 54.2 | 43 | 68.3 |  |
| G3 | 8 | 33.3 | 14 | 22.2 |  |
| Unknown | 3 | 12.5 | 6 | 9.5 |  |
| Laterality |  |  |  |  | **0.044** |
| Unilateral | 21 | 87.5 | 36 | 57.1 |  |
| Bilateral | 3 | 12.5 | 19 | 30.2 |  |
| Unknown | 0 | 0 | 8 | 12.7 |  |
| Preoperative tumor markers |  |  |  |  | 0.946 |
| CA125(U/ml) |  |  |  |  |  |
| Negative (<35) | 3 | 12.5 | 6 | 9.5 |  |
| Positive (>35) | 19 |  | 54 |  |  |
| 35~99 | 7 | 29.2 | 12 | 19.0 |  |
| 100~499 | 7 | 29.2 | 19 | 30.2 |  |
| ≥500 | 6 | 25.0 | 23 | 36.5 |  |
| Unknown | 1 | 4.2 | 3 | 4.8 |  |
| CA199(U/ml) |  |  |  |  | 0.719 |
| Negative (<37) | 8 | 33.3 | 25 | 39.7 |  |
| Positive (>37) | 12 |  | 31 |  |  |
| 37~99 | 5 | 20.8 | 8 | 12.7 |  |
| 99~499 | 4 | 16.7 | 11 | 17.5 |  |
| ≥500 | 3 | 12.5 | 12 | 19.0 |  |
| Unknown | 4 | 16.7 | 7 | 11.1 |  |
| HE4 (pM) |  |  |  |  | 1 |
| Negative (<70) | 5 | 20.8 | 14 | 22.2 |  |
| Positive (≥70) | 13 |  | 43 |  |  |
| 70~499 | 12 | 50.0 | 27 | 42.9 |  |
| ≥500 | 1 | 4.2 | 16 | 25.4 |  |
| Unknown | 6 | 25.0 | 6 | 9.5 |  |

^a^：mean ± stdev.

Table S4. Treatment and survival outcomes of the 12 patients with recurrence.

| **Case** | **FIGO stage** | **Surgery** | **Residual disease** | **Cycle of chemotherapy after primary surgery** | **Treatment after recurrence** | **Chemotherapy regime after recurrence** | **Follow-up status** | **Survival time after recurrence** |
| --- | --- | --- | --- | --- | --- | --- | --- | --- |
| 1 | IIIB | Primary cytoreductive surgery | R0 | 8 | - | NA | Dead | 0 |
| 2 | IIB | Staging surgery | R0 | 6 | Clinical trial | Ahead-OC-301 | Dead | 7 |
| 3 | IIIC | Primary cytoreductive surgery | R0 | 8 | - | NA | Dead | 0 |
| 4 | IC | Staging surgery | R0 | 6 | - | NA | Alive | 2 |
| 5 | IIIC | Primary cytoreductive surgery | R0 | 6 | Chemotherapy | NA | Dead | 14 |
| 6 | IIIB | Primary cytoreductive surgery | R0 | 6 | Chemotherapy | NA | Dead | 2 |
| 7 | IV | Primary cytoreductive surgery | Rx | 6 | Surgery + chemotherapy | Docetaxel + Lobaplatin | Alive | 10 |
| 8 | IIIC | Primary cytoreductive surgery | R0 | 1+ | Chemotherapy | NA | Dead | 17 |
| 9 | IIIC | Primary cytoreductive surgery | R0 | 6 | Chemotherapy | Gemcitabine + Platinum;  Etoposide;  Taxanes + Platinum; | Dead | 28 |
| 10 | IIIC | Primary cytoreductive surgery | Rx | 6 | Chemotherapy | NA | Dead | 33 |
| 11 | IIIC | Primary cytoreductive surgery | R0 | 6 | - | NA | Dead | 28 |
| 12 | IIIB | Primary cytoreductive surgery | R0 | 6 | Chemotherapy | NA | Dead | 2 |

NA: not available

Ahead-OC-301: apatinib mesylate tablets fitting etoposide soft capsules versus paclitaxel weekly therapy in patients with recurrent ovarian cancer who failed platinum therapy: a randomized, open, controlled, multicenter, phase III clinical trial.
