# Supplementary figures and images for: An analysis of clinical characteristics and prognosis of endometrioid ovarian cancer based on the SEER database and two centers in China
Source: BMC Cancer. 2023 Jul 1;23:608. doi: 10.1186/s12885-023-11048-1 (PMC10314552; doi:10.1186/s12885-023-11048-1)

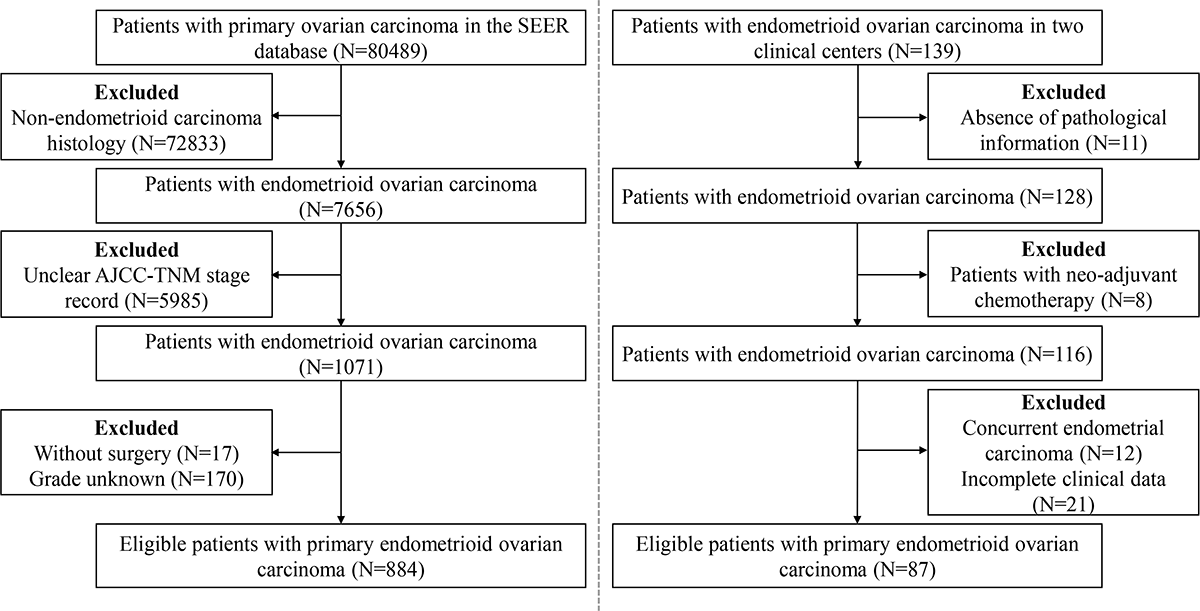

Supplement: Supplementary file 2 — Additional file 2: Figure S1. Flowchart of the selection for patients with endometrioid ovarian carcinoma. Figure S2. The calibration curves of one-year, three-year and five-year overall survival of the nomogram. Figure S3. Distribution of age, FIGO stage and grade of EOVC patients of the SEER database (N=884) and our centers (N=87). [file 12885_2023_11048_MOESM2_ESM.zip › Additianal file 2/Figure S1.tif]

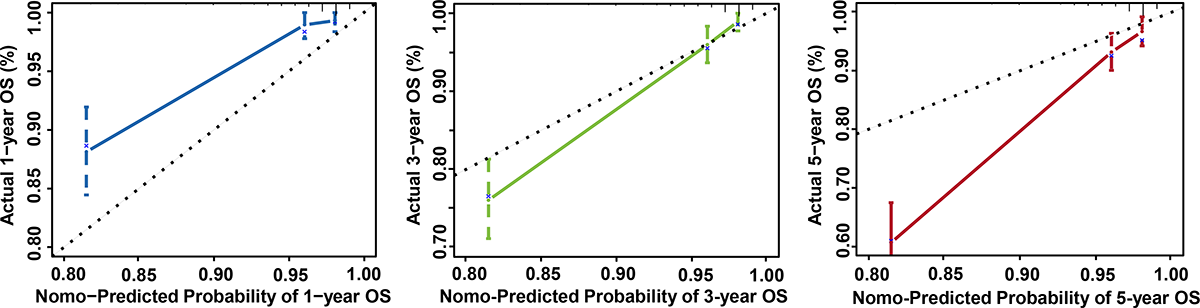

Supplement: Supplementary file 2 — Additional file 2: Figure S1. Flowchart of the selection for patients with endometrioid ovarian carcinoma. Figure S2. The calibration curves of one-year, three-year and five-year overall survival of the nomogram. Figure S3. Distribution of age, FIGO stage and grade of EOVC patients of the SEER database (N=884) and our centers (N=87). [file 12885_2023_11048_MOESM2_ESM.zip › Additianal file 2/Figure S2.tif]

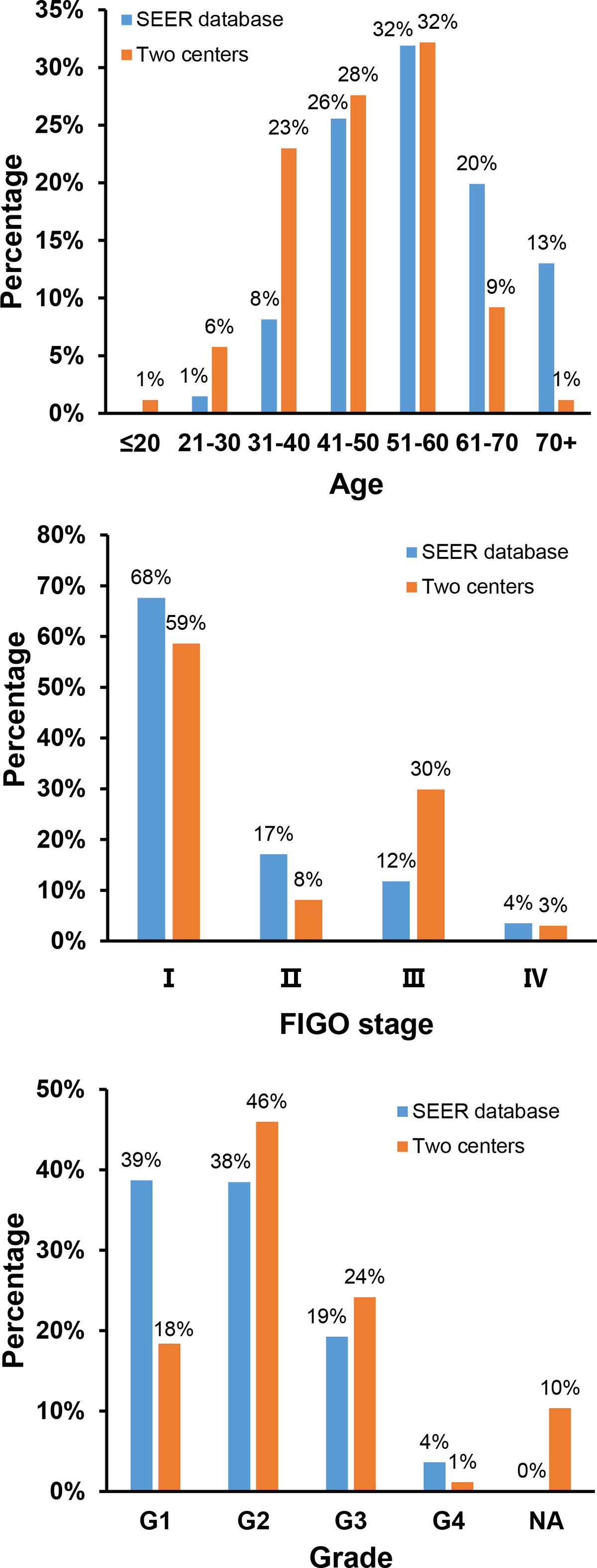

Supplement: Supplementary file 2 — Additional file 2: Figure S1. Flowchart of the selection for patients with endometrioid ovarian carcinoma. Figure S2. The calibration curves of one-year, three-year and five-year overall survival of the nomogram. Figure S3. Distribution of age, FIGO stage and grade of EOVC patients of the SEER database (N=884) and our centers (N=87). [file 12885_2023_11048_MOESM2_ESM.zip › Additianal file 2/Figure S3.tif]
